# Decolonising research methodologies: lessons from a qualitative research project, Cape Town, South Africa

**DOI:** 10.1080/16549716.2018.1561175

**Published:** 2019-01-21

**Authors:** Mpoe Johannah Keikelame, Leslie Swartz

**Affiliations:** Department of Psychology, Stellenbosch University, Cape Town, South Africa

**Keywords:** Indigenous, power, trust, culture and cultural competency

## Abstract

**Background**: It is becoming increasingly important for researchers to critically reflect on approaches that can have a positive impact on the health outcomes of indigenous people. Such issues are of great importance and perhaps of special relevance to researchers in the Global South, and to the African context in which we work.

**Objective:**To share some lessons learned from our fieldwork to contribute to current knowledge and conversations on decolonising research process.

**Methods**: We used an African lens to critically reflect upon some issues raised from individual interviews and focus group discussions with our participants which we deem to be important for consideration in a decolonising research process.

**Results**: The major issues that we raise are about important structures such as power, trust, cultural competence, respectful and legitimate research practice and recognition of individual and communities’ health assets in a decolonising research process.

**Conclusions**: Our paper argues for alternative approaches which are culturally appropriate for health research and for improved health outcomes of marginalised groups. In addition, we argue that participatory and transformative research methods which recognises individual and communities’ assets are needed. We hope that the lessons that we share in this paper can contribute towards a respectful and good research practice among the marginalised population groups in our context.

## Background

When indigenous people become the researchers and not merely the researched, the activity of research is transformed. Questions are framed differently, priorities are ranked differently, problems are defined differently, people participate on different terms. (Smith 1999, cited in Zavala [1, p. 59])

It is becoming increasingly important for researchers to critically reflect on approaches that can have a positive impact on the health outcomes of indigenous people. Such issues are of great importance and perhaps of special relevance to research in the Global South, and to the African context in which we work. According to Nhemachena et al. [2], research conducted among indigenous people in Africa has often not resulted in improved health outcomes of the researched. For these authors, research has in fact resulted in Africa suffering from a ‘resource curse of collectors and discoverers of African material resources, cultural artefacts and knowledge’ [p. 9].

A decolonising research methodology is an approach that is used to challenge the Eurocentric research methods that undermine the local knowledge and experiences of the marginalised population groups [2–5]. As stated by Goduka et al. in Khupe and Keane [3, p. 26], for research to be relevant and thus improve the quality of life of indigenous people, it should be driven by indigenous worldviews, cultural values and a language that is relevant to the indigenous group with whom research is undertaken. It should also be propelled by constructive discussions on knowledge systems and how these systems restrain and exclude other forms of knowledge, and the kind of actions needed for these systems to be more open and integrated [4]. For Zavala [1], researchers should note that decolonising research is not as much about the method, but more about the spaces that can enable the research process – and that through this process, researchers’ identities also become reshaped or transformed. Scholars writing in this field argue that researchers should use an indigenous lens in all phases of the project to scrutinise the choice of theoretical frameworks and methodologies they use and how research findings can be translated into actions that promote social justice [4,5]. On the other hand, Louis (cited in Keane et al.) [6, p. 14], caution that research that has negative health outcomes for the researched should not be conducted.

## Colonialism in South Africa

Colonialism has been reported globally as one of the key determinants of health of indigenous population groups [7], while post-colonialism is about the ongoing struggle to address the impact of the injustices of colonialism on indigenous people [4]. In terms of colonisation in South Africa, Oliver and Oliver [8, p. 4–8] report that the country had four types of colonisation: (i) unofficial colonisation by the Black people from the north; (ii) official colonisation from the south by the Dutch VOC; (iii) official colonisation by Great Britain; and (iv) internal colonisation from 1961 by the White Afrikaners which ended in 1994. According to these authors, the White Afrikaner apartheid rule was the severest of all types of colonisation due to its oppressive and exclusive policies. Despite the country’s changeover from apartheid to democracy, the major disparities in provision of healthcare still exists and has greatly affected the health and well-being of the majority of the low socio-economic population groups [9,10].

Though on paper there are several policies to redress these health inequities, there has been very little progress regarding the transformation of the healthcare system via the envisaged National Health Insurance system (NHI). The NHI system aims to prioritise healthcare services and to provide inclusive comprehensive public healthcare coverage and access to the healthcare services to all population groups in the country [11]. However, Rispel [12] and van Rensburg [13] warn that factors such as poor leadership, management and governance, lack of a fully functional district health system and political commitment to address the health workforce crisis may inhibit the successful implementation of the NHI system.

In this article, we use an African lens to highlight some important research process issues which came to the fore on conducting a qualitative study which explored perspectives and subjective experiences of adults who had epilepsy and their carers in an urban Xhosa speaking township in Cape Town, South Africa. We argue that structures such as power, trust, culture and cultural competence, respectful and legitimate research practice and recognition of individual and communities’ assets are important issues to consider when conducting research among the marginalised population groups.

## Background to the study

Our qualitative research project explored perspectives and subjective experiences of living with epilepsy and caring for people having the illness. The setting in which we conducted the study is an urban township in Cape Town, South Africa – and one of the first townships established under the oppressive laws of the apartheid system. This resulted in the marginalisation of the residents – a situation that remains unchanged even after democracy. As part of the study, we interviewed adult patients with epilepsy and their carers. Carers included family members, home-based carers who provide community-based care via home visits, and traditional healers. In South Africa, most of the population consult with these healers for their healthcare needs [14]. We are of the view that the lessons we share from our reflexive process will enable other researchers to learn from our experiences and that these can contribute to current knowledge and conversations in this field.

Although we use the term indigenous, we note from literature that there are controversies surrounding the definition of indigeneity in low- and middle-income countries (LMICs). Others, such as Ohenjo et al., as cited in Mohindra [15, p. 582], state that many Africans view themselves as indigenous due to their past experiences of colonialism. But Hillary Weaver [16] cautions that indigeneity is a very complex term which is the subject of controversy, and that it can have different interpretations. The author critically questions whether the term refers to issues such as race, ethnicity, tribal identity, cultural identity or other types of identity. Others such as Telles and Torche [17] are of the view that indigeneity cannot be understood through censuses because of their reliance on certain indicators. They caution against referring to all people as indigenous as this may risk ignoring the perspectives of the majority of the marginalised and those to whom indigeneity may be worthwhile. We are therefore of the view that future research should explore how the term indigeneity is understood and perceived by the researched and the researchers in our context.

## Our research experience and lessons for a decolonising research process

Drawing from her experience in the field, the first author, Keikelame [18], reflects on some of the challenges that she faced on conducting her research project on epilepsy, recently published in an article entitled: *The tortoise under the couch: An African woman’s reflections on negotiating inside-outsider positionalities and issues of serendipity on conducting a qualitative research project*. Keikelame is a Black African South African woman, but nevertheless not an insider to the cultural group she was studying. Despite that she is conversant with the groups’ spoken language, her positions often shifted between insider and outsider due to varied contextual issues that came to the fore during fieldwork. She uses a metaphor of a tortoise to share her critical reflections on some of these important lessons that emerged during her fieldwork which could be vital for a decolonising process. For her, an approach to a decolonising process should be paced slowly and should be characterised by commitment, courage and perseverance – like the tortoise's commitment. She shares some important issues that other researchers can learn from when doing research among the marginalised population groups: issues of language; of interpreters, translators and transcribers; of ethics; of religion and faith; of reciprocity; of unexpectedness; and of identity, age and gender.

In the section below, we discuss the five central tensions and structures which we deem to be of importance in decolonising research methodologies: (i) power; (ii) trust; (iii) culture and cultural competence; (iv) respectful and legitimate research practice; and (v) recognition of individual and communities’ assets. We use the pronoun ‘I’ to refer to the first author as she is the principal investigator, and the larger part of our discussion is based on her personal experiences, reflections and interactions in the field.

### Power

The notion of a *‘power with’* rather than *‘power over’* approach when conducting research among marginalised and vulnerable population groups is crucial because the approach puts emphasis on equal power sharing between researchers and the researched [19, p. 76]. Power can be influenced by the researchers’ outsider-insider positions as well as the experiences of colonialism on both the researcher and the researched [20]. It is deemed by Harrits [21] as a coercive structure that permeates all human relations. As reported by Darroch and Giles [22], a feminist lens may be useful to examine issues of power that are characterised by race, education and class. For these theorists, the researcher is not the sole producer of knowledge – but both the researcher and the researched make equitable and valuable contributions to the research process. They contend that issues of power should therefore be examined throughout the research process.

In our study, issues of power emerged from some stories told by our participants. From our focus group discussion with traditional healers, we learned that formal agreements such as the memorandum of understanding (MOU) and protection of traditional knowledge is crucial [23]. This concern about MOUs has also been raised by Alcock et al. [24] in their content-based literature review conducted in Canada. They concluded that MOUs are vital for engaging in legitimate and respectful research practice and assert that such a step is a form of empowerment of research partners and can also enhance sound collaboration through transparency of roles, expectations and actions. Other authors such as Alonso [25] state that such agreements are important because they are powerful tools with which people can fight against exploitation of their rights and to object to unfair research practices. Additionally, Masango [26, p. 76] reports that the World Intellectual Property Organization deems it important to protect traditional knowledge ‘from being exploited by appropriation for financial gains by third parties’. Masango [26] further states that protection of indigenous knowledge is crucial because traditional knowledge includes knowledge about the use of some plants, identification of medicinal properties in some of the plants and harvesting practices – this knowledge can therefore be protected within intellectual property rights. In addition, the design of MOU’s can be enabled by using strategies such as mediation and setting up formal partnerships that recognises the role of indigenous people from being ‘researched’ to ‘researchers’ [24].

### Trust

Trust is one of the important structures that underpin good research practice and a crucial indicator for sound relationships in a research process [27,28]. As reported by Moodley and Singh [27], colonialism and apartheid has perpetuated the lack of trust of scientists. The authors further point out that there is a need for a cultural revolution in health research which can be achieved through prioritising community engagement as a strategy to build trust in the research process. According to Liamputtong [28], trust building between the researcher and the researched in a decolonising research process is vital and development thereof should be based on values of respect, reciprocity, collaboration and co-operation. On the other hand, Abelson et al. [29] report that trust is one aspect that cannot be separated from vulnerability because the establishment of trust goes hand in hand with protecting the vulnerable against exploitation. For Boekraad [30], trust can be established by both indigenous and non-indigenous researchers. The author further states that non-indigenous researchers have played a positive role by engaging in productive and respectful research-related relationships based on trust. In other words, issues of trust and respect can therefore be established by indigenous and non-indigenous researchers who embraces these values in the research process.

### Culture and cultural competence

The difficulties surrounding the definition of the term Cultural Competence (CC) have been highlighted by Kleinman and Benson [31]. For them, this term is not about knowledge and skills, nor about the ‘do’s and don’ts’, because culture is not static and is not homogenous. Others, such as Beavis et al. [7], highlight that there is a need to think beyond cultural competence and to encourage critical consciousness when examining the social, political and historical issues. Some are of the view that continuous engagement with the researched can enable researchers to gain a deeper understanding of the culture and history of the study population [32]. For example, the first author (Keikelame) was invited by the chair of the Traditional Healers Organisation (THO) to attend provincial heritage celebrations. This invitation enabled her to learn some of the cultural ways through which the different traditional healers celebrate heritage, such as their styles of dancing and music. She jotted down the statement made by one of the key leaders of the Congress of Traditional Leaders of South Africa (CONTRALESA):

African researchers are the ones who can bring the true originality of the African traditional customs through their heritage…and heritage must not exclude culture, Ubuntu, nature and language. (Personal communication, CONTRALESA leader, 12 May 2013)

From this leader’s statement, we learned about some important aspects for consideration in a decolonising research process such as language, Ubuntu, culture, and respect for nature (the physical world). Ubuntu refers to an African ethic of interdependence and relatedness. Within the precepts of Ubuntu, personhood is understood in relational terms, as seen in the aphorism ‘A person is a person because of other people’. It stands in contrast to more individualistic ways of viewing personhood. According to Khupe et al. [33], research that is guided by Ubuntu can enhance trusting relationships and can also foster the notion of research *‘with’* the researched and not of research *‘on’* them. In terms of cultural heritage, Owusu-Ansa and Mji [34] reiterate that African scholars should be frontiers of their own cultural heritage and should engage in actions that empower and liberate Africans from poverty and social injustices of oppression.

In our analysis of one of the stories of our participants, we learned about the importance of paying attention to cultural beliefs in the research process. One of our participants had concerns about the loss of blood records and the repeated drawing of blood, often without his or her knowledge of the kind of blood tests that were to be done and reasons for taking blood [35]. There are different rumours and myths about blood that have been reported, such as stealing of blood and spreading of human immunodeficiency virus (HIV) [36]; and some beliefs that blood is related to the psyche and demonic spirits [37]. We are of the view that when analysing participants’ stories, it is important for researchers to bring to the fore these cultural issues and the meanings an interpretation attached to them. Writing about the importance of attending to issues of culture in a decolonising research, Tuhiwai-Smith [5] highlights that cultural beliefs, values, practices and norms should not be deemed as inhibitors of research. She emphasises that they should be an integral part of the indigenous research methodology and should be explicitly built into the methodology and reflected upon in a transparent way. This is quite important because, if these are ignored by the researchers, these may have an impact on the health outcomes of the researched or the research process itself.

### Respectful and legitimate research practice

The importance of clearer guidelines for conducting research among indigenous people cannot be over-emphasised. According to Mohindra [15,38], indigenous research practices need to be scrutinised to ensure that they are culturally appropriate and ethical for research conducted with and among indigenous people. Unethical research practices have been reported among the San people of southern Africa [39]. As a means to protect unethical practices among these groups, the South African San Institute and the leaders of the three San communities of the !Xun, Khwe and !Khomani who represent about 8000 people in South Africa developed the first San code of research ethics [39,40]. This code of ethics specifically highlights the four key ethical principles which underpin research among the San community: respect, honesty, justice and fairness, and care. It is envisaged that this code will ensure that the interests of the San communities are attended to by researchers [39].

Another critical issue reported in the San code of research is their concern about the use of scientific language. Similarly, in our project, we learned about the language barriers and how these may affect access to appropriate care and gaining informed consent to participate in research. During our recruitment phase, we faced challenges with our translated informed consent information leaflets which were translated by professional Xhosa-speaking translators. We learned from our local field interpreters that our professionally translated forms were not in the everyday spoken Xhosa language and may not be fully understandable to research participants. Although these local interpreters may have fewer formal qualifications than these professional translators, they speak the everyday spoken language of the researched, and thus would be vital in translations of these research protocols – an important aspect for decolonising research methodologies [5,18].

Regarding respect, we learned about the importance thereof in fostering collaboration between traditional healers and biomedicals. However, the healers with whom we interacted were of the view that there were some biomedical professionals who are disrespectful of their indigenous knowledge. In the process, Keikelame invited a leader of the local traditional healers’ organisation (THO) to attend a forum in which she presented findings from the interviews she had with them on their perspectives about epilepsy and collaboration with biomedicals. This leader openly raised concerns about exploitation of their indigenous knowledge in research:

Our concerns with research is that, we [traditional healers/leaders] willingly share our knowledge about the indigenous plants that we use to treat illnesses, but after the information has been given to researchers, we do not hear about the results and how plants have been processed into medicines; we do not see our names on the labels of the medicine bottles of the plants we have provided… (Traditional leader)

By contrast, we note from literature that there are some African scholars who reject working with these healers [41]. But Wreford, as cited in Bateman [42, p. 81], states that from her experience ‘traditional healers are open to collaboration…but biomedicals tend to close the door on their understanding and biomedical and traditional systems can work together in an inclusive and pragmatic way’. It is however, worthy of note that the emic and etic perspectives are always present in the research process. This is because the researchers’ own values direct the research approach, design, implementation, analysis, interpretation and reporting of results [Yin, 2010 cited in Olive, 43].

### Recognition of individual and communities’ assets

One of the key lessons that we learned from our project is the importance of research to recognise the individual and community health assets. These assets are defined as any factors or resources which enhance the ability of individuals, communities and populations to maintain and sustain health and well-being and assist in reducing health inequalities – and can operate at the individual, family, community and societal levels [44]. Sweet et al. [45] point out that a decolonising process is about reorientation from problematising indigenous people to a focus on their strengths, capacities and resilience – to a proper process in which time and opportunities to develop relationships and trust are created. According to Vaandrager and Kennedy [46], health assets are important resources which can foster individuals, communities and societies to engage in actions for social justice. In our project, we became aware of some of these assets at an individual level, such as self-advocacy, interpretation and networking skills. Here is an excerpt from Keikelame’s field notes:

One of my participants showed me her story published in the local newspaper where she talked about the difficulties of caring for her disabled partner who has epilepsy in a poorly structured one roomed shack with no electricity, sanitation and water. On my second visit to this family, I found that as a result of this action, she was able to access a three-roomed house with basic needs.

## Conclusion and recommendations

In this article, our aim was to share some of the lessons that we learned in our research process, and which we assume would be a useful contribution to informing the decolonising research methodologies process. Although we did not set out to use the indigenous methods per se, we found that on critical reflection from our different perspectives and orientations, we were able to gain insights into some of the important aspects that can be used to promote a culturally appropriate research practice.

Our central argument in this article is that issues of power, trust, culture and cultural competence, respectful and legitimate research practice and recognition of individual and communities’ assets are important structures to be considered in a decolonising process. Drawing from Smith’s (cited in Zavala [1]) quotation in the introductory section of this article, we are of the view that the transformation of research can happen when the researched and the researchers are involved from the initial development of the identification of the research problem, development of the research proposal, design and methodology and implementation in all phases of the project. In addition, active participation of the researched can enable collective ownership, collective data analysis, collective presentation and communication of findings [47]. We pointed out the importance of recognition of assets as one of the lessons that we learned in our fieldwork which we deem may be useful for decolonising research. The Asset-Based Community Development (ABCD) approach has been reported as an approach that can strengthen individuals’ and communities’ capabilities to become resilient [44,48].

In terms of ethical issues in a decolonising process, Sabati [49] highlights that research ethics need to be reframed in a way that can foster active institutional commitments to shift resources and research practices to forms of knowledge that are anti-colonial and that these should be central to how researchers engage with ethical issues. Our lessons further show that indigenous and non-indigenous researchers need to critically reflect on issues of culture to ensure that research among the marginalised is ethically and culturally appropriate. As reported by Gray and Oprescu [50], culturally appropriate research methods should be characterised by respectful relationships, open and respectful communication and dialogue.

Regarding indigenous knowledge, we learn from Braun et al. [51] that research among indigenous people often discredits their knowledge due to views that indigenous people are barbaric – and this may subject them to stigma, to vilifying their knowledge and using them as subjects of unethical practices as well as research. Despite this, we are of the view that a transformative process can address such negative attitudes and help to advance the understanding and the importance of indigenous knowledge in a decolonising research process.

In conclusion, the importance of reflexivity and self-reflexivity as a transformative approach in a decolonising process cannot be over emphasised. Stelmach [52] points out that non-Aboriginal researchers can engage in appropriate research if they turn the research ‘focus inward’ – by examining their own approaches throughout the research process, as well as the study design, interview questions, and data collection techniques, and by paying attention to their actual responses to participants’ comments. We are therefore of the view that even White researchers in our context can conduct appropriate research among the marginalised and vulnerable populations if they turn the research ‘focus inward’…as when the tortoise retracts its neck back into its shell. We trust that the lessons that we shared in this article will motivate other researchers to critically reflect on issues that may enhance a decolonising research process in the Global South and in our African context, and on how we, as researchers, can be empowered and transformed in the research process.

## Paper context

The importance of a need to transform research methods into those that can have improved outcomes on the health of indigenous people is needed. We provide some lessons from our fieldwork which can be used as alternative approaches from an African perspective which can advance our understanding of the methodological issues that can be considered in the decolonising research methodologies process in our context.
